# Inferring ontology graph structures using OWL reasoning

**DOI:** 10.1186/s12859-017-1999-8

**Published:** 2018-01-05

**Authors:** Miguel Ángel Rodríguez-García, Robert Hoehndorf

**Affiliations:** 1 Computational Bioscience Research Center, King Abdullah University of Science and Technology, Thuwal, 23955-6900 Kingdom of Saudi Arabia; 2 Computer, Electrical and Mathematical Sciences & Engineering Division, King Abdullah University of Science and Technology, Thuwal, 23955-6900 Kingdom of Saudi Arabia

**Keywords:** Ontology, OWL, Automated reasoning, Semantic similarity, Ontology visualization

## Abstract

**Background:**

Ontologies are representations of a conceptualization of a domain. Traditionally, ontologies in biology were represented as directed acyclic graphs (DAG) which represent the backbone taxonomy and additional relations between classes. These graphs are widely exploited for data analysis in the form of ontology enrichment or computation of semantic similarity. More recently, ontologies are developed in a formal language such as the Web Ontology Language (OWL) and consist of a set of axioms through which classes are defined or constrained. While the taxonomy of an ontology can be inferred directly from the axioms of an ontology as one of the standard OWL reasoning tasks, creating general graph structures from OWL ontologies that exploit the ontologies’ semantic content remains a challenge.

**Results:**

We developed a method to transform ontologies into graphs using an automated reasoner while taking into account all relations between classes. Searching for (existential) patterns in the deductive closure of ontologies, we can identify relations between classes that are implied but not asserted and generate graph structures that encode for a large part of the ontologies’ semantic content. We demonstrate the advantages of our method by applying it to inference of protein-protein interactions through semantic similarity over the Gene Ontology and demonstrate that performance is increased when graph structures are inferred using deductive inference according to our method. Our software and experiment results are available at http://github.com/bio-ontology-research-group/Onto2Graph.

**Conclusions:**

Onto2Graph is a method to generate graph structures from OWL ontologies using automated reasoning. The resulting graphs can be used for improved ontology visualization and ontology-based data analysis.

**Electronic supplementary material:**

The online version of this article (doi:10.1186/s12859-017-1999-8) contains supplementary material, which is available to authorized users.

## Background

An ontology is an explicit representation of a conceptualization of a domain [1, 2], and ontologies are widely applied in biology and biomedicine for annotation and integration of data [3]. The BioPortal ontology repository alone now lists over 500 ontologies [4], with several more ontologies under development. In the past, ontologies in biology were widely developed as directed acyclic graphs (DAGs) in which nodes stand for classes of entities within a domain, and edges for relations between these classes. For example, the classes *developmental cell growth* (GO:0048588), *cell growth* (GO:0016049) and *cell development* (GO:0048468) in the Gene Ontology (GO) [5] would be represented as nodes, and the relations between them by an edge from *developmental cell growth* to *cell growth* with an *is-a* label, and from *developmental cell growth* to *cell development* with a *part-of* label [6].

More recently, many ontologies are implemented in the Web Ontology Language (OWL) [7]. OWL is a formal language based on Description Logics [8] and offers a formal, model-theoretic semantics. Consequently, there have been several approaches for converting graph-based representations of ontologies into representations based on first order logic or description logic. For example, the OBO Relation Ontology [6] provides a systematic way to transform graphs into formal theories by giving explicit definitions for relations. Furthermore, approaches have been developed to convert graph-based representations of ontologies into OWL ontologies using an explicit translation relation [9, 10].

However, ontologies are not only used to express the knowledge within a domain but also for data analysis [3]. In particular, ontology enrichment analysis and semantic similarity measures are applied for predicting protein-protein interactions [11, 12], finding candidate genes of diseases [13–15] or classifying chemicals [16]. Most of these measures crucially rely on graph structures [17]. For example, the majority of semantic similarity measures used in biology are graph similarity measures [18], and ontology enrichment analysis utilizes the graph structure of ontologies to detect over- or under-represented classes [19, 20]. Consequently, there is now a gap between the increasingly more formal representation languages used for ontologies in biology and the analysis methods that utilize them, and a need to generate graph structures from ontologies that also take into account the semantics of the axioms in ontologies.

One of the standard reasoning tasks in OWL ontologies is the generation of the backbone taxonomy underlying an ontology [8] based on the axioms provided. This *classification* task is used to generate graphs in which subsumption (i.e., *is-a*) relations are expressed, but cannot easily be used to generate different types of edges, such as those labeled *part-of*, which represent axioms involving complex class descriptions [6]. In general, these edges can also not be created syntactically; an obvious example is a general concept inclusion axiom (i.e., an axiom in which a complex class description instead of a named class appears on both sides of a subclass axiom), in which axioms involving object properties cannot clearly be associated with a single class, or the inferences resulting from the use of inverse object properties or property hierarchies. While axioms in OWL may be arbitrarily complex and may not easily be representable in a graph-based form, they may imply axioms that can naturally be expressed in the form of a graph. For example, when nodes in a graph represent named classes, an axiom such as A or B SubClassOf: R some C cannot be represented (as A or B would not have a representation). However, this axiom implies that both A SubClassOf: R some C and B SubClassOf: R some C, and these inferences can be represented by two edges labeled R between A and C as well as between B and C.

Here, we describe a method to generate graph structures from OWL ontologies using only the semantic information (i.e., the axioms) contained in the ontologies combined with automated reasoning. We extend our previous work on visualizing ontologies in the AberOWL ontology repository [21] by improving our algorithm to generate sparser graphs (through the use of a transitive reduction) and making our conversion available as a stand-alone tool so that other researchers can integrate it in their analyses. Our method generates taxonomies as well as graphs containing other types of edges. We demonstrate that the graphs generated by our method outperform taxonomies and graphs generated using syntactic approaches when predicting protein-protein interactions through measures of similarity, demonstrating that our approach not only improves usability and representation of ontologies but also ontology-based data analysis methods. We implement our method in the Onto2Graph tool which is freely available at http://github.com/bio-ontology-research-group/Onto2Graph.

## Methods

### Ontologies

We obtained a list of all ontologies from the AberOWL ontology repository [22] to run our experiments. We downloaded all ontologies on 4 November 2015. We further perform a detailed evaluation on the Gene Ontology (GO) [5], and the GO extended with additional axioms and links to other ontologies, GO-Plus [23], also downloaded from the AberOWL ontology repository on 4 November 2015.

### Interaction datasets and functional annotations

For evaluation of the performance of different types of graphs in computing semantic similarity, we selected the Biological General Repository for Interaction Datasets (BioGRID) [24], which contains over one million protein-protein interactions and genetic interactions that occur in different types of organisms. Particularly, we selected the protein-protein interactions and genetic interactions occurring in fruitfly (*Drosophila melanogaster*), mouse (*Mus musculus*), nematode worm (*Caenorhabditis elegans*), yeast (*Saccharomyces cerevisiae*) and zebrafish (*Danio rerio*) to evaluate our results. We downloaded all interaction data from BioGRID on 29/11/2015.

As second interaction dataset, we identified GO annotations with the IGI (inferred from genetic interaction) and IPI (inferred from protein interaction) evidence codes. These annotations contain the interaction partner as part of the annotation, and we use these as a second interaction dataset for evaluation (separated into protein-protein interactions for the IPI evidence code and genetic interactions for IGI).

We obtained the GO annotations of proteins and genes from FlyBase [25], the Mouse Genome Informatics (MGI) database [26], WormBase [27], Saccharomyces Genome Database (SGD) [28], and the Zebrafish Information Network (ZFIN) [29]. We downloaded all GO annotations on 29/11/2015. Table 1 provides an overview over the datasets we use.

**Table 1:** Overview of the databases used in this work

Database	Species	Number of genetic interactions (IGI)	Number of physical interactions (IPI)
BioGRID	Fly	9978	37809
	Mouse	309	22914
	Worm	2330	6318
	Yeast	210791	127161
	Fish	70	214
GO	Fly	3151	2840
	Mouse	7996	12434
	Worm	2253	3205
	Yeast	3738	2415
	Fish	3623	720

### Onto2Graph

The Onto2Graph tool is developed in the Groovy language and implements the conversion algorithm (see Algorithm 1) to automatically transform OWL ontologies into graphs. Onto2Graph can generate graphs in different representation formats: RDF/XML [30], GraphViz [31], the OBO Flatfile Format [32], GraphML [33], and an output format used for the ontology enrichment tool OntoFUNC [34]. Onto2Graph uses the OWLAPI [35] to process ontologies and integrates the Elk reasoner [36], HermiT [37] as well as the structural reasoner that is part of the OWLAPI [35]. Output formats and reasoners can be selected as command line parameters and are generated using the Java Universal Network/Graph Framework (JUNG) [38].

### Visualizing graphs

In order to enable users to visualize the graphs, we generate graphs using OWLAPI’s structural reasoner and the Elk reasoner for all ontologies in AberOWL and store them in an OpenLink Virtuoso RDF store [39] for which we provide a public SPARQL endpoint at http://bio2vec.net/sparql/. Differences between syntactically generated graphs and graphs generated through the Elk reasoner can be retrieved through SPARQL queries. We further developed a visualisation environment to browse the structure of the graphs and analyse them easily. The visualization is based on LodLive project [40], and we modified the project so that it is possible to browse two graphs simultaneously for comparison. The resulting web interface is located in http://bio2vec.net/graphs/.

### Computing similarity and evaluation

We compute semantic similarity over the GO using the Semantic Measures Library (SML) [17]. We use the simGIC similarity measure [41] and Resnik’s measure [42] to compute pairwise semantic similarity between proteins within a species, using the best-match average as strategy to combine multiple pairwise similarity values. As the SML considers only subclass edges when computing semantic similarity, we rewrite other edge types generated through our algorithm as subclass edges before computing semantic similarity.

For each protein, we rank each protein by their similarity in descending order. Using our datasets of interactions as positive instances and all other pairs of proteins as negatives, we generate the ROC curves and compute the area under the ROC curve (ROCAUC) [43]. When comparing the difference between two ROC curves, we compute the difference in ROCAUC and perform a Wilcoxon rank sum test to determine whether the difference is significant [44].

## Results and discussion

### Converting OWL ontologies into graphs

We developed an algorithm (Algorithm 1) to transform OWL ontologies into multi-graphs using an automated reasoner that generates a proof for every edge included in the graph. The input of the algorithm is an OWL ontology with a set of object properties based on which edges in the graph are generated. Subclass (*is-a*) edges are created directly using an automated reasoner by classifying the ontology. For edges based on an object property *o*, however, such as a *part-of* edge, our algorithm identifies the most specific (existential) *o*-successor of a class *X* (an *o*-successor of node *n*_*s*_ is a node *n*_*t*_ in the resulting graph that should be connected through an edge labeled *o* to *n*_*s*_). For this purpose, the algorithm first identifies all candidate *o*-successors *P*_*X*_ of class *X* by querying for classes that are a subclass (or equivalent class) of o some X. It then queries each subclass *Y* of *X* for subclasses of o some Y to identify the candidate *o*-successors *P*_*Y*_ of *Y* (to improve performance of the algorithm, we only query all *direct* subclasses *Y* of *X*; if any subclass of *X* would be a candidate *o*-successor, then at least one direct subclass of *X* would also be a candidate *o*-successor). The direct *o*-successors of *X* are then classes that are candidate *o*-successors of *X* but not of any *Y*: 
1

**Algorithm 1:** Algorithm to generate a sparse graph representation of an ontology using an automated reasoner to interpret the ontology axioms. Any operation involving retrieving subclasses or direct subclasses (*subcl* and *direct-subclasses*) as well as classifying the ontology is performed using an automated reasoner.

Furthermore, to build a more concise graph while considering the semantics of the axioms involving object properties, we have added the option to perform a transitive reduction of the resulting graph over edges resulting from transitive object properties, subclass edges, and any combinations thereof.

The conversion is performed in two different steps (see Algorithm 1). In the first step, the algorithm processes the ontology and pre-computes the candidate *o*-successors of each class. In the second step, the *o*-successors are identified and added to the output graph as edges; if required, a transitive reduction is performed at this stage. The backbone of the graph is formed by the taxonomy of the ontology, i.e., the subclass and equivalent class relations between named classes, and we add the *o*-successors generated for each class: if *C* has an *o*-successor *D*, we generate an edge from *C* to *D* labeled *o*. The algorithm can generate multiple edges with different labels between the same nodes. For example, if *o*_1_ is a sub-property of *o*_2_ and an *o*_1_-labeled edge is generated between nodes *X* and *Y*, then our algorithm will also generate an *o*_2_-labeled edge between *X* and *Y* unless this edge is removed due to a transitive reduction.

We implement two versions of the algorithm, one in which all operations are performed semantically through an OWL reasoner, and another in which operations are performed syntactically by analyzing the expression of the axioms. When using OWL reasoning, we currently use either the Elk reasoner [45] or HermiT [37], and plan to support further reasoners in the future.

When analyzing the OWL axioms syntactically, instead of using the Elk reasoner, we use the OWLAPI [35] to obtain all asserted subclass and equivalent class axioms in the ontology; within these, we identify the axioms in which a single class is asserted to be a subclass or equivalent class to a class expression *C*_*exp*_. We then examine whether *C*_*exp*_ syntactically follows the pattern o some X to generate the candidate *o*-successors *X*.

**Fig. 1:**
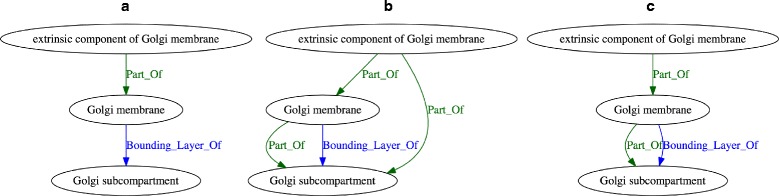
Example of inferring edges resulting from sub-property axioms and applying transitive reduction. **a** Syntactic reasoner, **b** Elk reasoner with t flag FALSE, **c** Elk reasoner with t flag TRUE

Figure 1 shows an example of three graphs generated by our approach from GO-Plus, first by using the syntactic approach to generating the graph (Fig. 1a), and then by utilizing the Elk reasoner without (Fig. 1b) and with (Fig. 1c) transitive reduction. Our approach is able to generate a graph-based representation based on any object property used within an ontology, and our method is particularly useful to generate these representations for transitive object properties.

### Semantically generating graphs improves performance of semantic similarity

Graph structures generated from ontologies are used for visualization as well as by several data analysis methods, and we evaluate the generated graphs by applying a (graph-based) semantic similarity measure to genes and gene products annotated with GO and evaluating the results for their performance in predicting protein-protein interactions and genetic interactions. To perform this evaluation, we select the GO-Plus ontology [23]. GO-Plus contains all the axioms in GO together with additional axioms, and may therefore be more suitable to demonstrate our approach as more edges can be inferred based on the additional axioms.

We generate two kinds of graphs from both of the ontologies: as a baseline, we generate graphs syntactically, i.e., based on the asserted axioms contained in each of the ontologies; and we generate graphs semantically by using our method with Elk reasoner. We also build graphs of different complexity. The first pair of graphs we generate contain only subclass relations but ignore all other object properties in the ontologies. The second kind of graph contains subclass relations and *part-of* relations, and the third kind of graph contains subclass, *part-of* and *regulates* relations. While GO contains additional object properties, we limit our analysis to *subclass*, *part-of* and *regulates* as these are the most frequently used object properties in GO. Table 2 shows the runtime of Onto2Graph when converting the GO-Plus ontology, as well as the runtime for computing the pairwise semantic similarity (using the simGIC measure) between all gene products in the mouse based on their GO annotations.

**Table 2:** Runtime of the Onto2Graph algorithm and semantic similarity computation over the GO-Plus ontology

Reasoner	Properties	Transitive reduction	Edges generated	Conversion runtime	Semantic similarity runtime
Elk reasoner	SubClassOf	True	146850	1 min 37 s	4 min 59 s
		False	146850	0 min 58 s	4 min 54 s
Elk reasoner	SubClassOf + PartOf	True	154583	10 min 50 s	5 min 42 s
		False	165457	10 min 37 s	5 min 44 s
Elk reasoner	SubClassOf + PartOf + Regulates	True	159261	16 min 34 s	6 min 21 s
		False	170112	16 min 10 s	6 min 15 s
Syntactic reasoner	SubClassOf	True	73692	0 min 48 s	3 min 25 s
		False	73692	0 min 46 s	3 min 30 s
Syntactic reasoner	SubClassOf + PartOf	True	82034	0 min 49 s	3 min 59 s
		False	82205	0 min 47 s	4 min 8 s
Syntactic reasoner	SubClassOf + PartOf + Regulates	True	85335	0 min 48 s	5 min 8 s
		False	85506	0 min 49 s	4 min 42 s

For the conversion and semantic similarity computation, we used a 3.20 GHz Intel i5-3470 CPU with 8 GB 1600 MHz DDR3 RAM and allowed Onto2Graph to use four threads

To employ these different graph representations of the ontologies in predicting interactions, we use functional annotations of proteins in fruitfly (*Drosophila melanogaster*), mouse (*Mus musculus*), worm (*Caenorhabditis elegans*), yeast (*Saccharomyces cerevisiae*) and zebrafish (*Danio rerio*) to compute pairwise semantic similarity over these graphs using the simGIC [41] semantic similarity measure. We use the similarity values to indicate interactions (either protein-protein interactions or genetic interactions) and evaluate the results using ROC analysis [43]. The results include the area under the ROC curve (ROCAUC) for each combination of the three generated graphs and the reasoners used to generate the graphs. We further perform a two-tailed Mann-Whitney *U* test to determine whether the observed differences in ROCAUC are significant and use Bonferroni correction [46] to adjust *p*-values for multiple testing. Table 3 summarizes our results. Full results are provided as Additional files 1 and 2.

**Table 3:** Summary of results obtained from graphs based on asserted axioms vs graphs semantically generated

Relationship	Species	Database	Type of interaction	AUC			SRvsELK(false)		SRvsELK(true)		ELK(false)vsELK(true)	
				SR	ELK (true)	ELK (false)	AUC difference	Two-tailed test	AUC difference	Two-tailed test	AUC difference	Two-tailed test
SubClassOf	Mouse	GA	IGI	0.88643	0.90463	0.90463	-0.01820	1.54×10^−05^	-0.01820	1.54×10^−05^	0	1
	Yeast	BG	Genetic	0.64086	0.651407	0.651407	-0.01055	2.93×10^−22^	-0.01055	2.93×10^−22^	0	1
		BG	Physical	0.70577	0.71460	0.71460	-0.00882	1.88×10^−10^	-0.00882	1.88×10^−10^	0	1
SubClassOf PartOf	Yeast	BG	Physical	0.70723	0.71559	0.71559	-0.00836	1.36×10^−09^	-0.00836	1.36×10^−9^	0	0.99984
SubClassOf PartOf Regulates	Mouse	GA	IGI	0.88979	0.90772	0.90767	-0.01793	1.61×10^−05^	-0.017988	1.71×10^−05^	5.10×10^−05^	0.98979
	Yeast	BG	Genetic	0.64785	0.65923	0.65923	-0.01138	1.22×10^−24^	-0.01138	1.20×10^−24^	− 2.00×10^−06^	0.99832
		BG	Physical	0.70944	0.71756	0.71755	-0.00812	3.66×10^−09^	-0.00811	3.83×10^−09^	1.00×10^−05^	0.99394

We have generated three types of graphs: a) only taking into account *SubClassOf* relationship; b) considering *SubClassOf* and *PartOf*; and c) finally *SubClassOf*, *PartOf* and *Regulates*. The table shows the performance obtained from predicting genetic and physical interactions for those model organisms that the statistical tests provide a significant results

We find that performance in predicting both protein-protein interactions and genetic interactions generally improves when using graphs generated by the Elk reasoner compared to graphs generated syntactically. While the increase in ROCAUC is not very large, it is, however, significant for several of our evaluation datasets. For example, for genetic interactions in yeast, we observe an increase of 0.011 AUC, which is significant (*p*=1.2×10^−24^, Mann-Whitney *U* test).

**Fig. 2:**
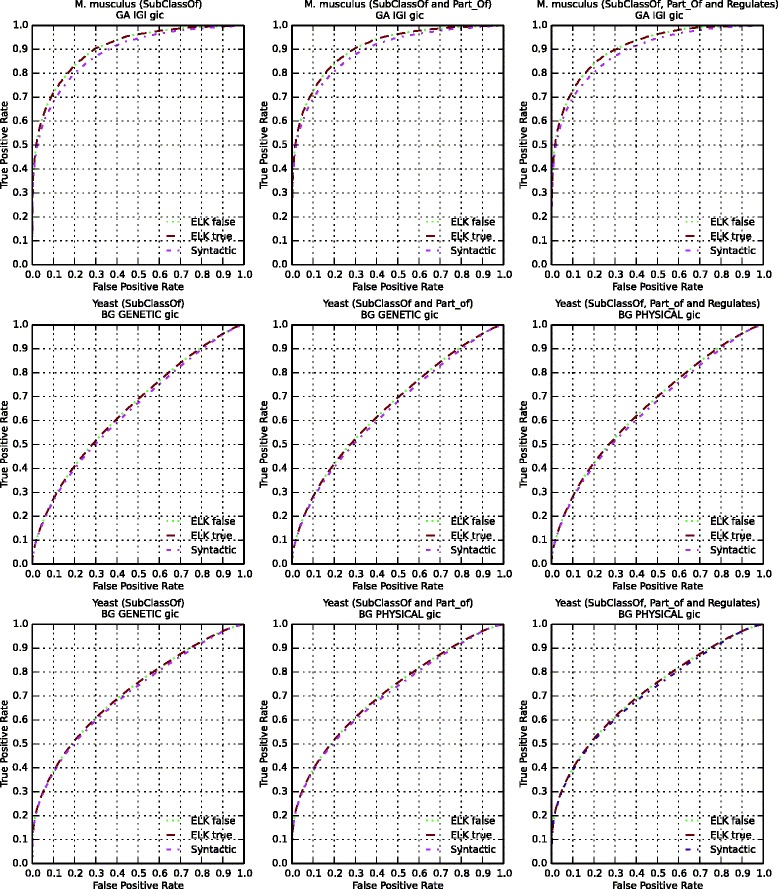
ROC Curves for predicting genetic interactions. We compare the performance of predicting genetic interactions using graphs generated from Gene Ontology Plus and the annotations available from Gene Ontology Annotation and BioGRID database. The green line refers to the performance obtained from the graph generated semantically without transitive reduction, brown with transitive reduction, and the pink line refers to the graph generated syntactically

Furthermore, if we compare the Elk-generated graphs with transitive reduction to Elk-generated graphs without transitive reduction, we also observe a slight increase in ROCAUC for predicting genetic interactions in yeast (0.2×10^−5^, Mann-Whitney *U* test). Generally, we observe a small but significant performance increase in most of our evaluation datasets, thereby demonstrating that our approach can generate graphs that may be better suited for biological data analysis than graphs generated using the asserted axioms in ontologies alone. The Fig. 2 shows a selection of the evaluation sets for which we observe significant improvement of ROCAUC.

## Conclusions

We developed the Onto2Graph conversion algorithm and tool that enables users to convert ontologies into graphs efficiently and utilizes an automated reasoner to infer edges in an ontology graph based on the ontology’s deductive closure. The tool integrates two different ways to perform this conversion, by using OWL reasoning and by syntactically analyzing the ontology axioms. The Onto2Graph tool can output graphs generated from OWL ontologies in several file formats which can then be used for ontology-based data analysis, such as semantic similarity or ontology enrichment analysis.

We demonstrated that the graphs generated by Onto2Graph can outperform graph structures generated syntactically or based on the ontology’s taxonomy alone when applied to computation of semantic similarity and prediction of protein-protein interactions. While the observed differences are small, our results nevertheless demonstrate how inclusion of more information that is already present within ontologies can contribute to biological discovery.

A major limitation of our current approach is the reliance on a single (existential) pattern to generate edges while many ontologies now use more complex axioms. While the Onto2Graph method can be applied to other relational patterns that should represent an edge within a graph, we did not implement this due to the computational costs involved in using arbitrary OWL axiom patterns [8]. In the future, the graph generated by our approach can also be used to infer additional edges used to build knowledge graph embeddings [47], and therefore contribute to applications of machine learning with ontologies.

## Additional files

Additional file 1 Comparison of syntactic and semantic reasoning. Supplementary file 1 contains full comparison results for syntactically generated graphs against graphs generated through automated reasoning. (XLSX 35 kb)

Additional file 2 Comparison of semantic reasoning with and without transitive reduction. Supplementary file 2 contains full comparison results for semantically generated graphs with and without transitive reduction. (XLSX 66 kb)
